# Editorial: Insights in aquatic physiology: 2021

**DOI:** 10.3389/fphys.2023.1202759

**Published:** 2023-04-26

**Authors:** Pung-Pung Hwang, Anna Di Cosmo, Silvia Franzellitti

**Affiliations:** ^1^ Institute of Cellular and Organismic Biology, Academia Sinica, Taipei, Taiwan; ^2^ Department of Biology, University of Naples Federico II, Naples, Italy; ^3^ Animal and Environmental Physiology Laboratory, Department of Biological, Geological and Environmental Sciences (BiGeA), University of Bologna, Ravenna, Italy

**Keywords:** aquatic animals, osmoregulation, acid-base regulation, respiration, behaviors

In recent years, scientists have made exceptional achievements that have led to major advancements in the fast-growing field of Aquatic Physiology. This editorial initiative, led by Prof. Andreas Fahlman, the previous Specialty Chief Editor of the Aquatic Physiology Section, is focused on the new insights, novel developments, current challenges, latest discoveries, and future perspectives in the field of Aquatic Physiology. Distinguished and active researchers have been invited to identify the greatest challenges in the sub-disciplines of Aquatic Physiology and offer solutions to address those challenges. This article Research Topic covers the basic mechanisms of osmoregulation, acid-base regulation, respiration, the nervous system, endocrines, behaviors, and digestion at the molecular, cellular, and biochemical levels, and further extends the discussion to aspects of evolutionary physiology. These articles will inspire, inform, and provide new direction to researchers in the field.

Aquatic animals must maintain ionic and acid-base homeostasis in their body fluids to ensure the normal operations of cellular activities and physiological processes. This mechanism is a basic platform for studying environmental adaptation, pollution toxicology, stress biology, and evolutionary physiology of aquatic animals, and further provides important information to improve management of aquaculture practice. Life in fresh water (FW) is osmotically and energetically challenging for living animals because they need to continuously take up Na^+^ (and other ions) from a salt-dilute environment. Our understanding of Na^+^ uptake mechanisms in invertebrates is much poorer than that in fishes. To this end, Lee et al. proposed the Na^+^/H^+^ antiporter (NHA) as a possible candidate for the Wieczorek Exchanger in many crustaceans and insects, which is hypothetically driven by apical proton pump V-type H^+^ ATPase (VHA). More studies are awaited to explore the Na^+^ uptake of NHA and other potential Wieczorek Exchangers. In the case of teleosts, which are the most studied fish group, Shih et al. reviewed the recent progress and clarified the related debates in their Na^+^ uptake mechanisms. FW teleosts absorb Na^+^ from the environment mainly via Na^+^/H^+^ exchanger 3 (NHE3)-mediated and Na^+^-Cl^-^ co-transporter (NCC) mediated mechanisms in gill/skin ionocytes, and they rely on NHE for a majority of Na^+^ uptake, probably due to a favorable NH_4_^+^ gradient that efficiently drives NHE. NHEs are considered the major acid-secreting transporters in seawater (SW) fishes as well as in FW ones. NHE3 is the most studied NHE isoform, while NHE2 and its function has never been clarified. According to Liu et al., NHE2 is localized at the basolateral membrane of fish ionocytes and appeared to facilitate acid and salt secretion in sea water (SW) medaka, providing a new insight into the role of NHE2 in SW osmoregulation. Gill (or skin) ionocytes are important sites for fish iono- and osmo-regulation. Different types of ionocytes expressing distinct sets of ion transporters were identified by Inokuchi and her colleagues (Inokuchi et al.) in tilapia (*Oreochromis mossambicus*) gills, providing a platform to explore the plasticity in ion transport functions and ionocyte morphologies in fish when coping with salinity challenges.

In addition to the gills, the kidney is an essential organ participating in fish osmoregulation. Recent progress in the identification and functional analyses of relevant ion transporters in the kidney of FW and SW teleosts were reviewed by Kato et al. These transporters are involved in the secretion of sulfate and magnesium ions (by the proximal tubules in SW), the reduction of urine volume (by the collecting ducts in SW), and the excretion of water through hypotonic urine (by the distal tubules and collecting ducts in FW). The kidney also plays a major role in osmoregulation of the few euryhaline elasmobranchs. For examples, in the euryhaline stingray that has acclimated to FW, the kidney, rather than gills, is the major organ responsible for NaCl reabsorption and dilute urine production. Aburatani et al. explored the roles of Na^+^-K^+^-Cl^-^ cotransporter 2 (NKCC2) and Na^+^/K^+^-ATPase (NKA) in NaCl and urea reabsorption (associated with dilute urine production) in the early distal tubule in the bundle zone of the Japanese red stingray (*Hemitrygon akajei*) kidney. Upregulation of the genes encoding NKCC2 and NAK found in FW-acclimated individuals is proposed as an advantageous feature that facilitate acclimation to a wide range of salinities, which might have allowed the batoids to expand their distribution towards low salinity habitats. The gastro-intestinal tract is the major organ for water drinking and absorption in fishes acclimation to SW. Grosell et al. compared different methods, whole-animal and isolated-tissue oxygen consumptions and the estimates of ATP consumption, to determine the metabolic cost of osmoregulation in the marine fish (*Opsanus beta*). The theoretical estimates of esophageal and intestinal osmoregulatory costs were in close agreement with direct measurements on isolated tissues, suggesting that these tissues amount to ∼2.5% of standard metabolic rates. The whole animal measurements were variable between fish and is not suited to detect the relatively modest metabolic cost of osmoregulation. The antennal gland is also an important osmoregulatory organ, particularly in terrestrial crabs. In the review by Tseng et al., species with higher NKA activity in the antennal gland showed a lower urine-hemolymph ratio for Na^+^ concentration under hypo-osmotic stress, suggesting a correlation between the structural and functional differences in gills and lung-like structure among crabs. This finding provides new insights into the evolutionary physiology of crab osmoregulation.

Secreting the excess acid that results from metabolic acidosis or an acidified environment is another essential task for aquatic animals to maintain their body fluid homeostasis. Acid secretion mechanism in aquatic animals is known to interact with the mechanisms involved in ion transport and ammonia excretion as well as to affect energy metabolism. It has been proposed that acid secretion mechanisms in fishes, as well as in mammals, is tightly controlled by hormones. Lin et al. reported a novel action of vitamin D (VD), a well-known calciotropic hormone, on zebrafish (*Danio rerio*) acid secretion. Signaling of VD and VD receptors upregulated acid secretion through stimulating the related acid-secreting transporters. Meanwhile, cephalopods live a lifestyle with high energy expenditure, which is fueled exclusively by protein diets, thus resulting in high production of ammonia as metabolic waste. According to Lin et al., benthic octopuses with lower aerobic respiration rates utilize both active and Na^+^-driven secondary transport mechanisms, while pelagic squids, with higher aerobic respiration rates, prefer active NH_4_^+^ and H^+^ transport mechanisms that consume ATP intensively. Therefore, diverse strategies were adopted by cephalopods to fit their different lifestyles.

It is critically important for fish to regulate and control their ventilation and maintain adequate water flow to match branchial gas transfer with their metabolic needs under different environmental or physiological situations. Perry et al. reviewed the control and consequences of ventilatory adjustments, the chemoreceptor cells and the mechanisms used to sense O_2_ and/or CO_2_ in zebrafish larvae. This review on zebrafish larvae paves the way to study fish respiratory physiology using advanced molecular physiological approaches that have been recently established in the species. A further study on fish respiratory physiology was done by Reed and Jonz. All the receptors implicated in the neurotransmission or neuromodulation associated with O_2_ sensing in the gills, including serotonergic, cholinergic, purinergic, and dopaminergic signaling, were summarized, but the receptor characterization at the cellular level remains to be done.

Behaviors are the outcomes of physiological performance that are integrated by the nervous system. Fu et al. summarized recent studies regarding the effects of internal and external stimuli on fish behaviors and proposed that studying behaviors can be an alternative way to conduct environmental and physiological assessments. Investigations into fish stress coping strategies, which are involved in endocrine control, physiological regulation, and behavioral response, are important because the related knowledge can improve fish welfare and promote the sustainable development of aquaculture. In the study by Zeng et al., active coping flounder increased prolactin-mediated ionocyte differentiation to deal with low-salinity, while passive coping flounder employed a passive strategy of reducing gill ionocytes number. Finally, the study by Moffatt et al. was extended to understand the mechanisms underpinning fish digestion and growth performance. The experiments with omeprazole, a gastric proton pump inhibitor, highlighted the importance of stomach acidification in digestion and growth and present a novel way of determining the cost of gastric digestion.

This Research Topic provides some new insights into our understanding of the basic physiology in aquatic animals and further suggests new directions for future studies.

